# Novel compound heterozygous *CDH23* variants in a patient with Usher syndrome type I

**DOI:** 10.1038/s41439-019-0037-y

**Published:** 2019-01-28

**Authors:** Satomi Okano, Yoshio Makita, Akihiro Katada, Yasuaki Harabuchi, Tomohiro Kohmoto, Takuya Naruto, Kiyoshi Masuda, Issei Imoto

**Affiliations:** 1 Hokkaido Asahikawa Habilitation Center for Disabled Children, Asahikawa, Japan; 2 Education Center, Asahikawa Medical University, Asahikawa, Japan; 3 Department of Otolaryngology-Head and Neck Surgery, Asahikawa Medical University, Asahikawa, Japan; 4 Department of Human Genetics, Graduate School of Biomedical Sciences, Tokushima University, Tokushima, Japan; 5 Risk Assessment Center, Aichi Cancer Center Hospital, Nagoya, Japan

**Keywords:** Disease genetics, Genomics

## Abstract

Usher syndrome type I (USH1) is characterized by congenital, bilateral, profound sensorineural hearing loss, vestibular areflexia, and adolescent-onset retinitis pigmentosa. Here, we report a 12-year-old female patient with typical USH1. Targeted panel sequencing revealed compound heterozygous variants of the *Cadherin 23* (*CDH23*) gene, which confirmed the USH1 diagnosis. A novel NM_022124.5:c.130G>A/p.(Glu44Lys) was identified, expanding the mutation spectrum of *CDH23*.

Usher syndrome (OMIM #276900) is an autosomal recessive disorder characterized by hearing loss and subsequent onset of retinitis pigmentosa. The frequency of Usher syndrome has been estimated to be 3.2–4.4 per 100,000 people in Europe and the United States^1^. This syndrome is classified into three subtypes based on the severity and onset of hearing loss^2^. Type I is the most severe form of deafness and blindness in humans. Profound hearing loss develops in infancy, accompanied by vestibular areflexia, followed by pigmentary degeneration of the retina around 10 years of age^3^. Until now, six genes are known to be responsible for Usher syndrome type I (USH1), which includes *MYO7A* (MIM 276900), *USH1C* (MIM 276904), *CDH23* (MIM 601067), *PCDH15* (MIM 605514), *SANS* (MIM 606943), and *CIB2* (MIM 614869)^4^. Proteins encoded by these genes are essential for the development and maintenance of the inner ear, and play a crucial role in the development of hair cells^5^. However, the roles of these genes in eyesight are still elusive. *CDH23* on 10q21 encodes a transmembrane Ca^2+^-dependent adhesion protein, cadherin 23 (CDH23), with cadherin-like domains, that is responsible for the diverse phenotypes of both nonsyndromic autosomal recessive deafness-12 (DFNB12) and Usher syndrome^6^. Herein, we report the case of a Japanese girl who developed USH1 with novel compound heterozygous variants in exon 3 and in the splice donor site within intron 10 of *CDH23*.

The patient is a 12-year-old Japanese girl who is the third child of non-consanguineous parents with no family history of deafness or blindness (Fig. 1a). There was no clinical history of maternal infections during pregnancy. She was born via vaginal delivery without asphyxia at 39 weeks of gestation, and her birth weight was 3612 g. During the neonatal period, her Moro reflex was absent, which indicated vestibular dysfunction. However, nystagmus was not observed. She visited our hospital at the age of 5 months because her parents noticed that she did not react to sounds. Her auditory brainstem response did not show any evoked responses below 100 decibel (dB) on both sides (Fig. 1b). Computed tomography and magnetic resonance imaging revealed that there were no structural abnormalities in her ears. A caloric test indicated semicircular canal paralysis. Her deafness was considered to be congenital and stable. Upon completion of a 20-month hearing aid trial without improvements, she received a cochlear implant in the right ear at an age of 2 years and 5 months. After the implantation of her cochlear implant, her aided hearing threshold level was 35 dB. She started walking at the age of 3. Her language function developed poorly in spite of her using the well-adjust cochlear implant and receiving special support from her school for the deaf. At present, she uses sign language for communication. At the age of 12 years, she complained of difficulty in sign communication at night. She developed tunnel vision, followed by rapidly progressive visual impairment, and eventually was diagnosed with retinitis pigmentosa.

**Fig. 1:**
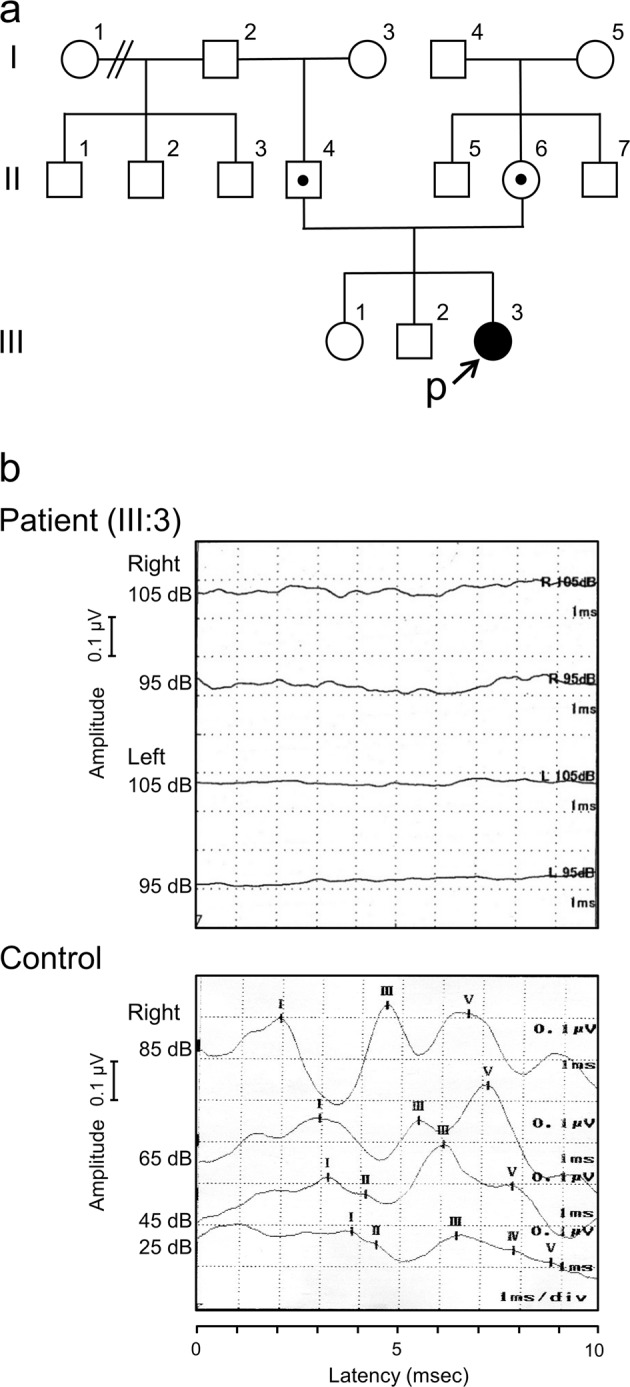
**a** Family pedigree. The proband is indicated by an arrow (P). The patient had no family history of hearing loss or retinitis pigmentosa. **b** Absence of all waves in auditory brainstem response recording even at 105 dB in both ears of the patient (III:3) at the age of 1 year and 4 months compared with a healthy control (below) indicates severe bilateral hearing loss

After written informed consent was obtained from her parents, a genetic analysis was performed. Commercially available genetic testing for deafness provided by BML, Inc. (Shibuya-ku, Tokyo, Japan) covering 154 loci in 19 genes showed a negative result. We then conducted a targeted panel sequencing (TPS) for the targeted exons of 4813 disease-related genes using a Trusight One Sequencing Panel (Illumina, San Diego, CA, USA), and an MiSeq sequencer (Illumina), followed by analysis using our pipeline for NGS data as described previously^7–9^. The ethics committees of Tokushima University approved the study. Sequence variants with higher allele frequencies (i.e., >0.01) in the following databases were excluded to identify presumably pathogenic single nucleotide variants: 1000 Genomes Project database (http://www.1000genomes.org), National Heart, Lung, and Blood Institute Grand Opportunity (NHLBI GO) Exome Sequencing Project (ESP6500, http://evs.gs.washington.edu/EVS), Human Genetic Variation Database (http://www.genome.med.kyoto-u.ac.jp/SnpDB) and integrative Japanese Genome Variation Database (iJGVD, https://ijgvd.megabank.tohoku.ac.jp). TPS revealed compound heterozygous variants in *CDH23* (NM_022124.5): one is paternally inherited c.130G>A in exon 3, and the other is maternally inherited c.945+1G>T in intron 10 (Fig. 2). Both of these variants were confirmed by trio Sanger sequencing. No other possibly pathogenic variants or gross deletions were detected in the coding regions of other five USH1-related genes (*MYO7A*, *USH1C*, *PCDH15*, *SANS*, and *CIB2*) in the panel for TPS. The splice site variant in intron 10 has been already registered in ClinVar (http://www.ncbi.nlm.nih.gov/clinvar/) as a pathogenic variant for USH1 (RCV00150272.1). Although c.130G>A/p.(Glu44Lys) variant has been listed neither in the human genome mutation database (HGMD, Professional 2017.4; http://www.hgmd.org/) nor ClinVar, we inferred it as pathogenic from the results of an in silico analysis. Glu44Lys was observed in the first cadherin domain (cadherin domain 1) of CDH23 and is the substitution of the highly conserved 11th glutamic acid (Glu) in the cadherin domains, which has been reported to lead to a weakness in binding with Ca^2+^ and altered protein conformation^10^, indicating this Glu to be indispensable for the protein function of CDH23, although this residue is outside of three highly conserved calcium-binding motifs (LDRE, DXNDN, and DXD)^1^. Pathogenic missense variant in the 11th glutamic acid (Glu) in the cadherin domain 3 [p.(Glu247Lys)] has been reported in the USH1 case^11^. Taken together, the patient was diagnosed with USH1 caused by one known and one novel variant of *CDH23* in a compound heterozygous state based on the results of this molecular diagnosis^12^.

**Fig. 2:**
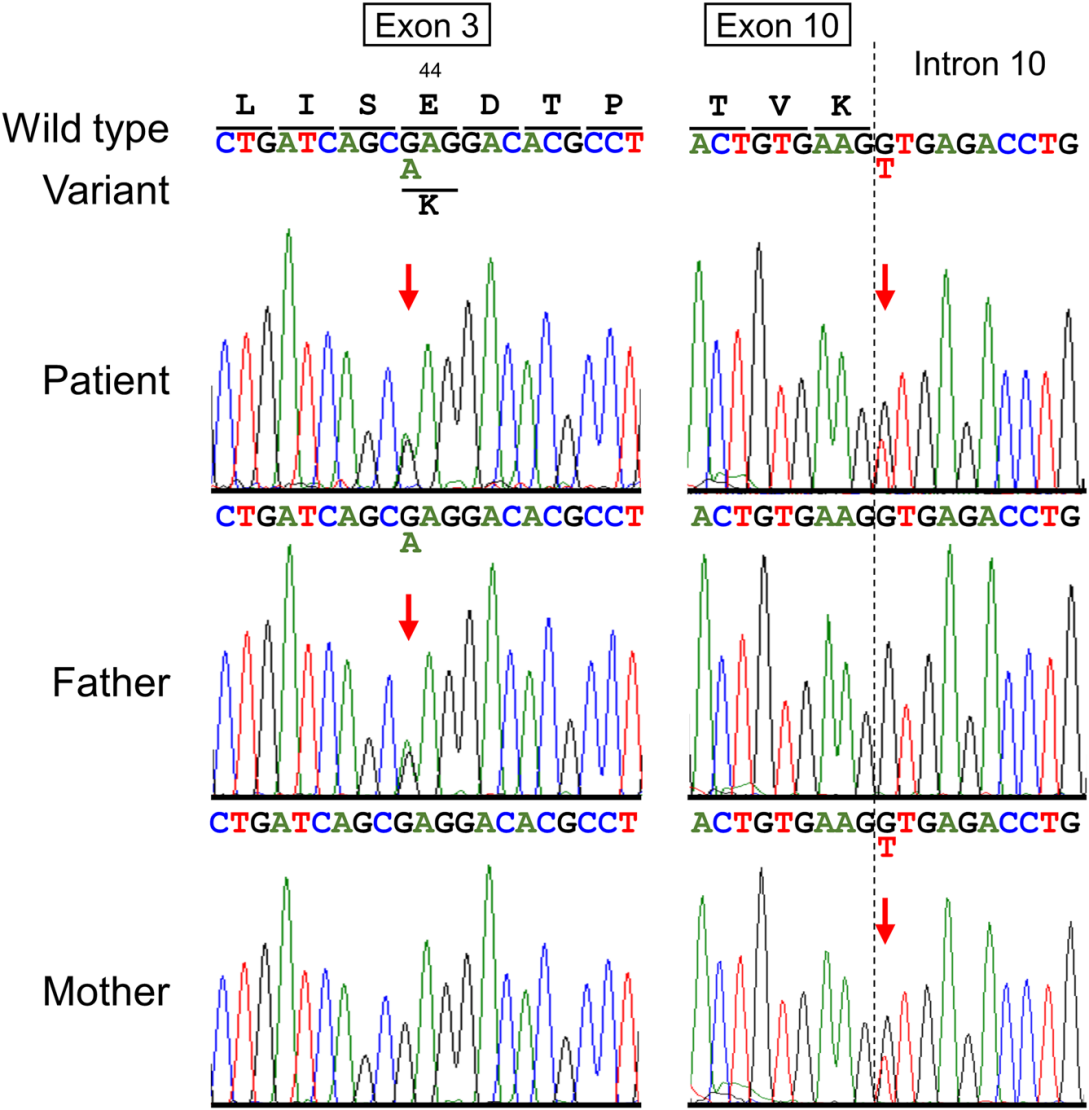
Partial sequence chromatograms for *CDH23* in the patient and both parents. DNA and corresponding amino acid sequences of the wild-type and mutant *CDH23* alleles are shown. Red arrows denote the sites of heterozygous sequences

In the inner ear, the mechanical forces of sound waves are transduced into electrochemical signals to be transmitted through the acoustic and equilibrium pathway. This transducer is contained in a bundle of stereocilia at the top of hair cells. The CDH23 protein localizes to the upper part of tip-link filament of crosslinking stereocilia, which is thought to gate the mechanoelectrical transduction channel^12,13^. A previous study has demonstrated that a *Cdh23* mutant mouse model had disrupted stereocilia organization, and that the protein encoded by *Cdh23* was a critical component of hair bundle formation^14,15^.

*CDH23* is responsible for the diverse phenotypes of both nonsyndromic DFNB12 deafness and USH1. DFNB12 is associated with *CDH23* missense mutations that are presumed to be hypomorphic alleles with sufficient residual activity for retinal and vestibular function, but not for auditory cochlear function (DFNB12 allele). In contrast, homozygous nonsense, frameshift, splice site, and some missense mutations of *CDH23*, all of which are presumably functional null alleles, cause USH1D (USH1D allele)^6,12^. In individuals with *CDH23* compound heterozygotes, the DFNB12 allele has been reported to be phenotypically dominant to an USH1D allele^6^. Therefore, the p.(Glu44Lys) observed in our case is likely to cause the null protein function of CDH23 possibly through a weakness in binding with Ca^2+^ and altered protein conformation, although the codon 44 is outside of three highly conserved peptide sequences (LDRE, DXNDN, and DXD), which directly bind to the calcium ion^6,10^. Similar pattern of *CDH23* compound heterozygotes, one substitution of the 11th Glu in the cadherin domain 3 [p.(Glu247Lys)] and one non-sense mutation [p.(Glu2554X)] has been reported in the patient with USH1^11^. Taken together, a combination of pathogenic/likely pathogenic *CDH23* variants in a compound heterozygote, one known USH1-causing splice-site variant and one possibly USH1-causing novel missense variant, is supposed to cause USH1 in our case. The pathogenic variants of causative genes, including *CDH23*, in a compound heterozygous state in Usher syndrome has been reported^11,12,16^. Combination of one missense and one splice-site *CDH23* variants has also been previously reported in patient with sector retinitis pigmentosa^17^. To identify compound heterozygous *CDH23* variants in USH1 similar to those in our case, a wide-range analysis using TPS may be useful.

In conclusion, we identified pathogenic novel compound heterozygous *CDH23* variants in a Japanese patient with USH1. The results matched the clinical symptoms of Usher syndrome and helped in genetic counseling. The limitation of this report is that the cause–effect relationship of observed *CDH23* variants was not well established. Further studies including a functional analysis and accumulation of more cases are needed to elucidate the pathogenesis of each *CDH23* variant.

## Data Availability

The relevant data from this Data Report are hosted at the Human Genome Variation Database at: 10.6084/m9.figshare.hgv.2519
